# Disseminated cystic echinococcosis presenting as orbital swelling in a child — case report and literature review

**DOI:** 10.1007/s15010-026-02743-9

**Published:** 2026-05-13

**Authors:** Jonathan Remppis, Mariya Gosheva, Robert Rottscholl, Hendrik Rosewich, Sabine Bélard, Marija Stojković

**Affiliations:** 1 Neuropediatrics, General Pediatrics, Diabetology, Endocrinology and Social Pediatrics, University Children’s Hospital Tübingen, Tübingen, Germany; 2 Institute of Tropical Medicine, University of Tübingen, Tübingen, Germany; 3 German Center for Infection Research (DZIF), Partner Site Tübingen, Tübingen, Germany; 4 Centre for Ophthalmology, University of Tübingen, Tübingen, Germany; 5 Institute for Pathology and Neuropathology, University of Tübingen, Tübingen, Germany; 6 Department of Infectious Disease and Tropical Medicine, Heidelberg University Hospital, Heidelberg, Germany; 7 Department of Neuropaediatrics, University Hospital Göttingen, Göttingen, Germany; 8 German Center for Child and Adolescent Health (DZKJ), Partner Site Göttingen, Göttingen, Germany

**Keywords:** Echinococcosis, Hydatid disease, Cyst, Child, Orbit, Spillage

## Abstract

**Background:**

Cystic echinococcosis (CE) is a parasitic disease that commonly affects the liver and lungs. Orbital involvement is an unusual presentation, particularly in non-endemic areas, and poses diagnostic and therapeutic challenges.

**Case Presentation and Literature Review:**

We report the case of a five-year-old girl from Syria who presented with orbital swelling and was ultimately diagnosed with disseminated CE. The diagnosis was delayed, and management was complicated by intraoperative cyst rupture and spillage. Surgical resection combined with antiparasitic therapy resulted in regression of the cysts. A scoping review of the literature identified 15 reports of orbital CE in children, most of them from endemic countries.

**Conclusion:**

CE should be considered in children presenting with cystic lesions and a history of migration from endemic areas. Early involvement of a CE reference center is essential to prevent complications and to optimize management.

## Introduction

Cystic echinococcosis (CE) is a zoonotic parasitosis caused by the tapeworm *Echinococcus granulosus.* Humans become accidental intermediate hosts after ingesting eggs, with parasites spreading via the bloodstream and forming hydatid cysts in various organs. Cysts most commonly develop in the liver, followed by the lungs, but can also locate at any other anatomical location. Clinical symptoms depend on the site and size of the cysts and potential complications which comprise cysto-biliary or cysto-bronchial fistula, bacterial cyst infection and cyst rupture [1, 2]. Management is guided by radiologic staging and comprises antiparasitic pharmacotherapy, surgery, percutaneous interventions and watch and wait strategies [3, 4]. CE is associated with poverty and causes substantial morbidity in endemic areas in South America, the Middle East, parts of sub-Saharan Africa, Asia, and eastern Europe. In high-income countries there are only sporadic infections. In 2023, 113 CE cases were reported in Germany, the majority in migrants from low-income countries [5, 6].

Here, we report the case of disseminated CE presenting as orbital swelling in a five-year-old girl with a migration background from Syria.

### Presentation of the case

**Fig. 1:**
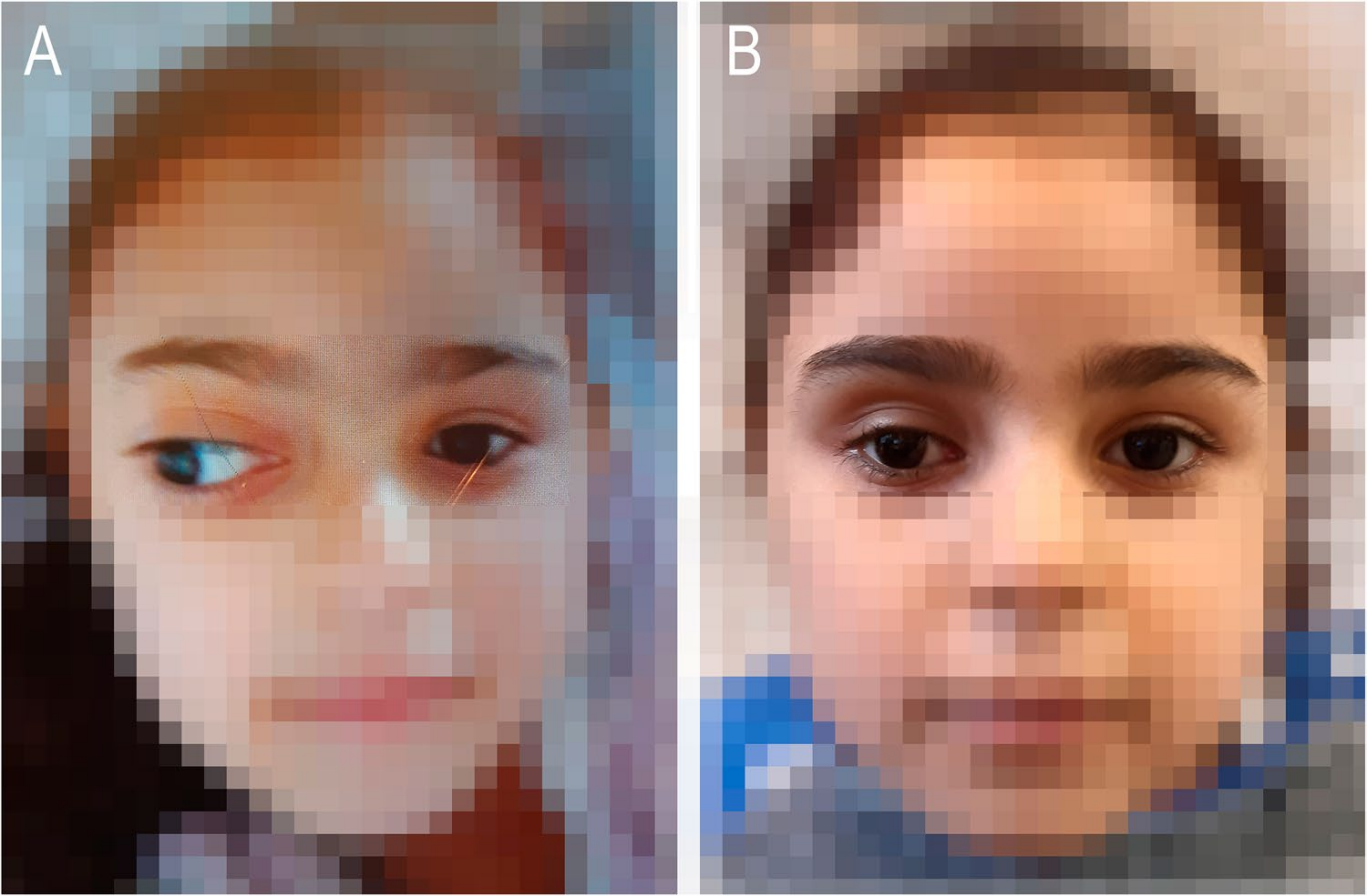
Right-sided orbital swelling due to a hydatid cyst. **a** before surgery **b** after surgery

In 2024, a five-year-old girl presented to the ophthalmology department because of right sided exophthalmos that had persisted for more than six months (Fig. 1a). The girl had moved from Syria to Germany with her family four months earlier. In Syria, a magnetic resonance imaging (MRI) scan of the head had been performed showing an orbital cyst of unknown origin. Due to the planned relocation to Germany, no further diagnostics or treatments had been pursued. Repeated nausea and vomiting were reported as additional symptoms. No other pre-known medical conditions or interventions were reported.

**Fig. 2:**
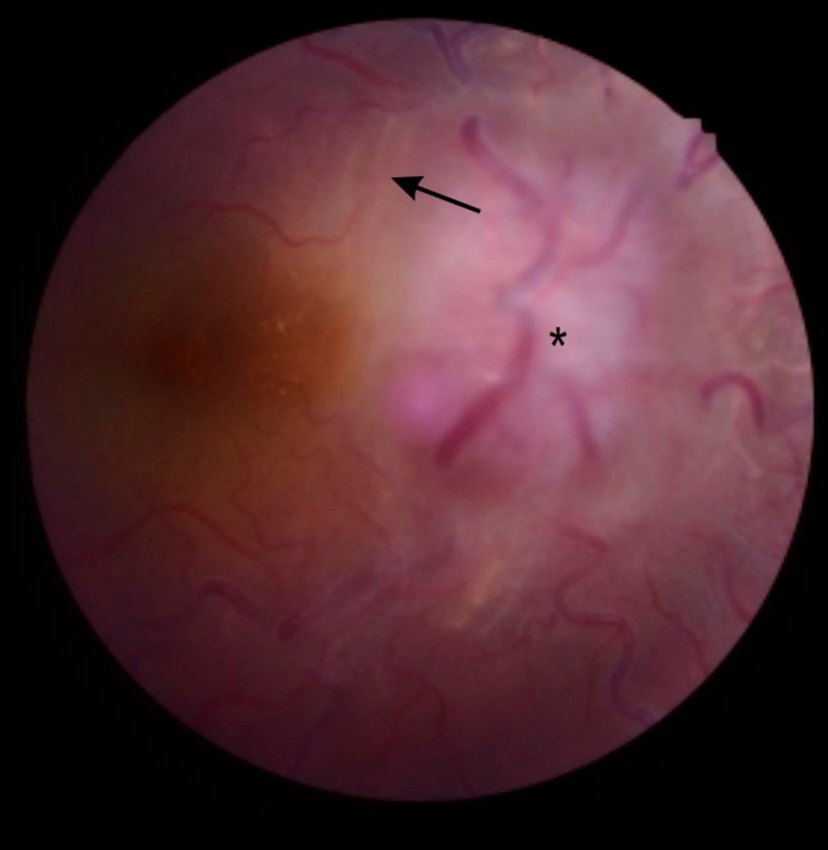
Fundus examination of the right eye showing papilledema (asterisk), choroidal folds (arrow) and subretinal fluid

Ophthalmologic examination of the right eye revealed reduced visual acuity (hand motion), exotropia with restricted motility in all directions, and a relative afferent pupillary disorder. Hertel exophthalmometry showed an exophthalmos of 9 mm on the right side. Fundoscopy demonstrated papilledema, choroidal folds and subretinal fluid (Fig. 2).

**Fig. 3:**
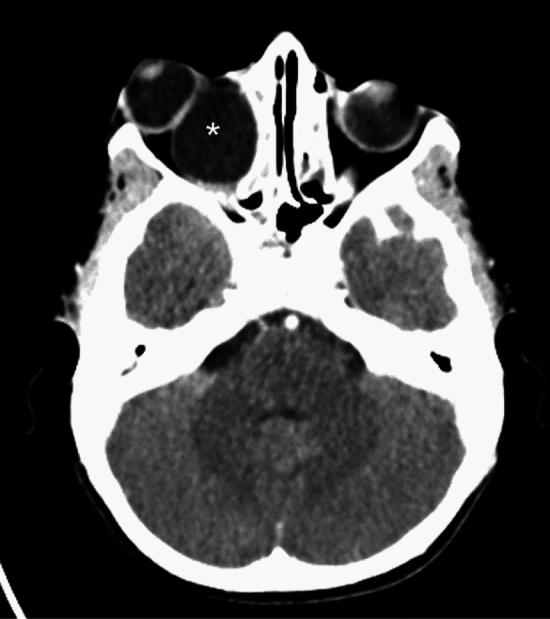
Computed tomography scan of the head, performed prior to surgery. The asterisk indicates the well-defined hydatid cyst in the right orbit

Due to papilledema and vomiting, intracranial hypertension was suspected and a computed tomography (CT) scan of the head was performed. While no signs of intracranial hypertension were detected, the scan revealed a well-defined cyst in the right orbit (Fig. 3).

**Fig. 4:**
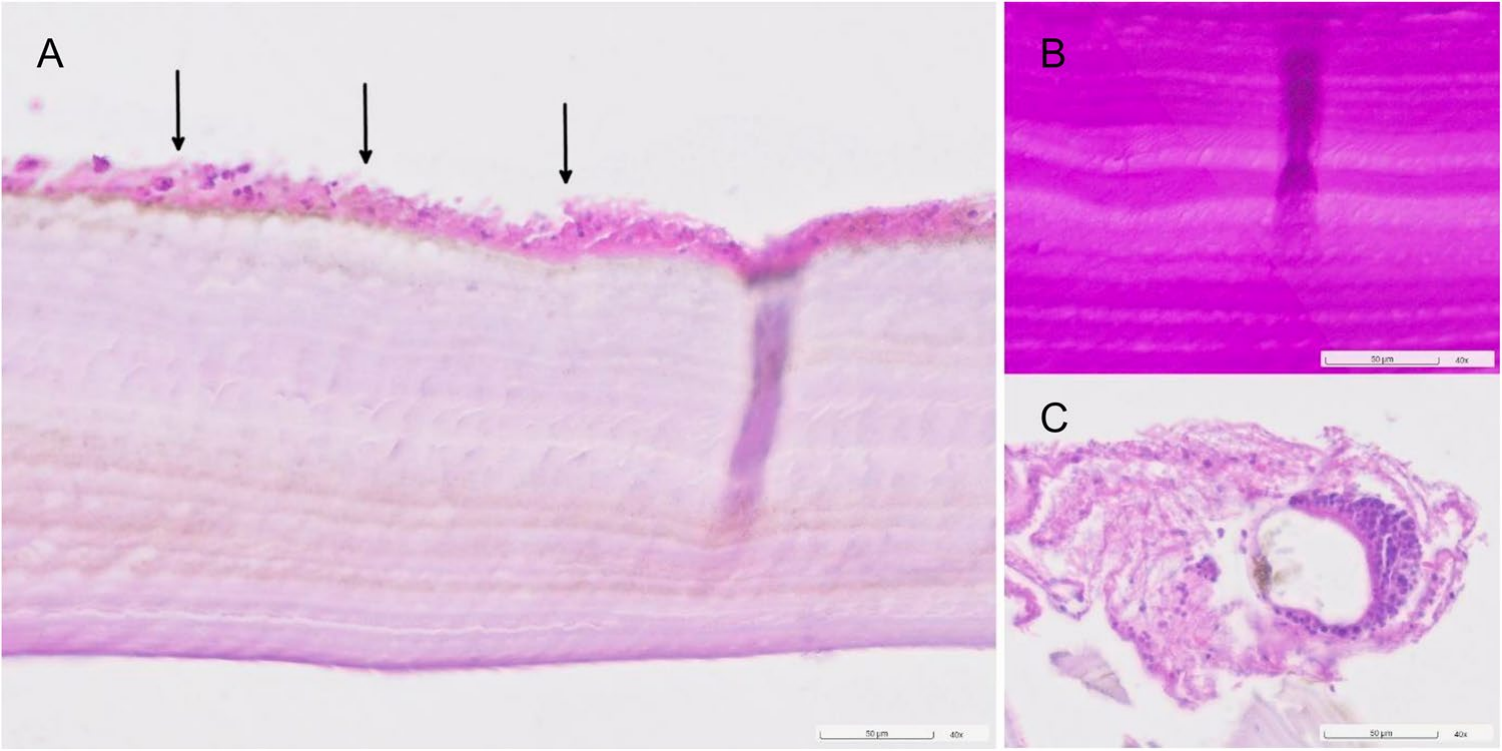
**a** Histologically, the resection specimen (hematoxylin–eosin stain, H&E) showed classical features of *E. granulosus* with a bilayered cyst wall with an outer laminated membrane and an inner germinal membrane (arrows). **b** Periodic acid–Schiff (PAS) staining showed the characteristic intense purple staining of the outer laminated membrane. **c** Serial section (H&E) with protoscolex. All images taken at × 40 magnification

The case was discussed in two interdisciplinary pediatric tumor boards and as the etiology of the cyst remained unclear, surgical resection with subsequent histological examination was decided. During surgical resection, the cyst ruptured spontaneously, releasing clear fluid that spilled into the orbital cavity. Histopathological examination of the resection specimen revealed membranous and cystic structures characteristic of *E. granulosus* (Fig. 4).

Due to suspected CE additional imaging to screen for further hydatid cysts was performed. Ultrasound showed cysts of the liver, both kidneys and the pectoral soft tissue. Chest x-ray showed bilateral pulmonary cysts. Serological testing included ELISA (Echinococcus granulosus and Echinococcus multilocularis IgG ELISA; Bordier Affinity Products, Crissier, Switzerland) and immunoblot analysis (Echinococcus Western Blot IgG; Biorepair.com Labordiagnostika GmbH, Sinsheim, Germany) which showed a serological pattern consistent with CE. In consideration of all findings, a diagnosis of disseminated cystic echinococcosis was established, and molecular analyses were not required.

**Fig. 5:**
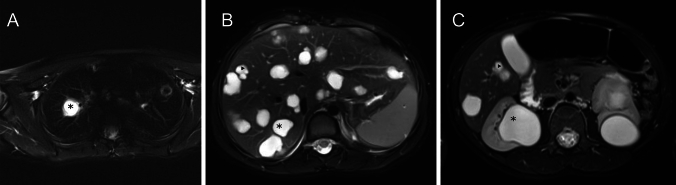
T2-weighted magnetic resonance imaging, performed prior to initiation of albendazole treatment showing multi-organ involvement with cysts in WHO stages CE1 (asterisks) and CE2 (arrowheads). **a** bipulmonary cysts **b** multiple hepatic cysts **c** bilateral kidney cysts

In consultation with the national clinical reference center for CE, post-exposure prophylaxis with praziquantel (40 mg/kg as a single daily dose) was initiated three days after surgery because of the intraoperative spillage. Prior start of albendazole treatment, a whole-body MRI was performed to better visualize and stage all cysts and rule out extensive lung involvement, given the potential risk of albendazole-induced cyst rupture into the bronchi. Staging revealed predominantly CE1 cysts and some stage CE2 cysts, according to WHO classification (Fig. 5) [4].

While the initial blood count had shown no abnormalities, the patient developed a transient eosinophilia (max. 18.8%) one week after surgery. After two weeks, the patient was discharged with albendazole (10 mg/kg per day in two divided doses), which was continued for 12 months. Praziquantel was discontinued after 14 days. Follow-up included repeated sonography and an MRI after 12 months, showing transition of all cysts into stage CE3a and no signs of orbital relapse. Ophthalmologic follow-up showed a complete restitution of the visual acuity, eye motility and pupillary function, and disappearance of the papilledema. The patient adhered well to the long-term albendazole therapy. Serial monitoring of blood counts and liver enzymes revealed no hematologic or hepatic adverse effects.

### Literature review

For a scoping review of the literature on orbital CE in children, we searched PubMed for articles published 2006 or later. Key search terms were: “child”, “pediatric”, “cystic echinococcosis”, “hydatid disease”, “eye,” “orbit” and “ophthalmology”. There were no language limitations. The search identified 25 reports which were screened by title and abstract. Twenty eligible articles underwent full text screening. Finally, 15 publications that included original reports of orbital CE in 22 children < 18 years were included (Table 1). The reported signs and symptoms were similar to those in our case, including mostly painless proptosis, reduced visual acuity, impaired ocular motility, and papilledema. Some children also presented with conjunctival chemosis, eyelid edema or eosinophilia. All reported CE cases were confirmed histologically, while positive serology was documented in only one case. Involvement of other organs was observed in a single child. Cyst rupture during surgical resection occurred in three cases. No recurrence of CE was reported; however, the duration and conditions of follow-up investigations varied considerably and in many reports, follow up after hospital discharge was either not performed or not reported. All but one of the cases were diagnosed and treated in countries endemic for CE.

**Table 1:** Pediatric cases of orbital cystic echinococcosis reported in the literature

Authors, publication year	Country	Pediatric cases	Age/sex	CE Serology	Other organs involved	Surgery	Anthelmintic treatment ^a^	Recurrence (follow up duration)
Yan et al., 2015 [7]	China	1	11y / f	NR	NR	Cyst resection, site irrigation	NR	No (NR)
Ozdemir et al., 2024 [8]	Turkey	1	2y / m	NR	No	Cyst aspiration	Albendazole	NR
Benazzou et al., 2010 [9]	Morocco	7	11y /m 6y / f 2y / m 7y / f 6y / m 3y / f 9y / m	Pos (n = 1); neg (n = 4); NR (n = 2)	No	Puncture & irrigation (n = 4); cyst rupture (n = 2); removal in toto (n = 1)	Mebendazole (n = 3); albendazole (n = 2); NR (n = 2)	No (3y, n = 5); NR (n = 2)
Papathanassiou et al., 2008 [10]	Greece	1	7y / f	Neg	Lung and liver	None	Albendazole	No (4y)
Kahveci et al., 2012 [11]	Turkey	1	6y / f	Neg	No	Cyst aspiration, injection, resection	Albendazole	NR
Lentzsch et al., 2016 [12]	Germany	1	5y / f	Neg	NR	Cyst resection	Albendazole	NR
Chtira et al., 2019 [13]	Morocco	1	3y / f	NR	No	Cyst puncture, removal of endocyst, site irrigation	Albendazole	No (2y)
Kumar et al., 2023 [14]	India	1	8y / m	NR	No	Cyst resection	Albendazole	NR
Alabdullah et al., 2025 [15]	Syria	1	10y / m	NR	No	Cyst puncture, removal of endocyst, site irrigation	Albendazole	NR
Awad et al., 2025 [16]	Egypt	2	11y / m 13y / m	NR	NR	Cyst aspiration, removal of endocyst	NR	No (1 – 5y, n = 2)
Saravi et al., 2016 [17]	Iran	1	12y / m	NR	NR	Cyst resection	NR	NR
Cruzado-Sánchez et al., 2017 [18]	Peru	1	10y / f	Neg	No	Cyst resection	None	No (1y)
Yurt et al., 2011 [19]	Turkey	1	3y / m	Neg	No	Cyst resection in toto	None	No (2y)
Gokhale et al., 2012 [20]	India	1	10y / f	NR	No	Cyst rupture, cyst resection, site irrigation	Albendazole	NR
Handor et al., 2013 [21]	Morocco	1	12y / NR	NR	NR	Cyst resection in toto	NR	NR
Presented case	Germany	1	5y / f	Pos	Liver, lung, kidneys, soft tissue	Cyst resection with accidental cyst rupture	Praziquantel Albendazole	No (1y)

*CE* cystic echinococcosis, *y* years, *f* female, *m* male, *NR* not reported

^a^ reported duration: between 2 weeks and 6 months

## Discussion

Pediatric CE rarely presents with orbital swelling as the leading symptom; thus, our case of an orbital cyst revealing disseminated CE in a child represents a rarity, particularly outside endemic areas. One of the diagnostic challenges is that CE cysts can mimic various conditions that are more common in low-prevalence countries, such as congenital malformations, benign tumors or cancer [3]. In our case, even though the diagnostic workup took place at a tertiary academic treatment center that holds experts of various medical fields, the suspicion of CE was raised only after surgical resection, on the base of histopathological examination. Because an infectious etiology of the orbital cyst was not initially suspected, pediatric infectious diseases and tropical medicine specialists, who might have accelerated the CE diagnosis, were not involved prior to the surgery. For the same reasons, neither additional imaging apart from the head nor *Echinococcus* antibody testing was done. While the latter is rarely positive in isolated orbital cysts and thus cannot rule out CE, the positive immunoblot would probably have led to an earlier diagnosis in our case of disseminated CE. In summary, the reported case points at a lack of awareness regarding CE in low-prevalence countries; CE must be considered a differential diagnosis in all cystic lesions detected in migrants from CE-endemic countries, not only in the liver or lungs, but also in other organs. CE screening in patients with orbital cysts should include ultrasound and serology; however, the limitations of both modalities must be considered. When CE is suspected, CT and orbital MRI are essential for diagnostic confirmation.

Challenges in the management of CE comprise lack of expertise in surgical or percutaneous treatment methods outside of specialized treatment centers, long distances between these centers, as well as language barriers and non-adherence to long-term antiparasitic treatment. In addition, there is a lack of evidence regarding the optimal duration of antiparasitic treatment. While albendazole is recommended for 3–6 months in uncomplicated hepatic cysts, patients with cysts at other or rare locations, multi-organ involvement or cyst rupture require individualized treatment [1–4]. Accordingly, the length of albendazole therapy varied considerably across the individual case reports included in our literature review. Our patient received a combination of praziquantel and long-term albendazole, based on WHO and other recommendations for the management of intraoperative spillage [1, 2]. As shown by our literature review, cyst rupture rarely occurs when orbital CE is treated in endemic areas, probably due to heightened awareness, earlier diagnosis and more experience in the surgical treatment. Spillage prophylaxis is a CE-specific surgical procedure comprising surgical field protection with pads soaked in 20% sodium chloride on top of pads moistened with normal saline and creation of a closed system during cyst evacuation [1, 3]. This critical step should always be performed if CE cannot be ruled out. In our case, the intraoperative spillage could have been prevented by earlier suspicion of CE.

Once the diagnosis of CE was established, subsequent interdisciplinary management proceeded effectively in our case. Guided by the CE reference center, the patient was treated and followed up close to the patient’s residence. Long-term follow-up is of paramount importance as it enables the treatment to be adjusted according to cyst activity. Once all cysts have reached an inactive stage, monitoring should continue for five years. Our case highlights the critical importance of specialized centers in ensuring state-of-the-art CE management in low-prevalence countries. Through telemedical consultation and teleradiological interpretation of MRI studies, expert guidance for the treatment of cystic echinococcosis can be provided effectively without requiring the patient’s physical presence.

## Conclusion

Our case illustrates the challenges in the diagnosis and management of CE in a very-low-prevalence country. In children with unclear cystic lesions and migration history, CE must be included in the differential diagnosis. Diagnostic workup should include radiological screening for further cysts in the liver and lungs, and early consultation of a CE reference center to guide management.

## Data Availability

No datasets were generated or analyzed during the current study.

## References

[1] Stojkovic M, Gottstein B, Weber TF, Cystic JT, Farrar J, Garcia PJ, et al. Manson’s tropical diseases. Curr Opin Infect Dis. 2023;36(5):318–25. 37578473 10.1097/QCO.0000000000000962 PMC10487362

[2] WHO guidelines for the treatment of patients with cystic echinococcosis. Geneva: World Health Organization. 2025. Licence: CC BY-NC-SA 3.0 IGO. 40690566

[3] Stojkovic M, Weber TF, Junghanss T. Clinical management of cystic echinococcosis: state of the art and perspectives. Curr Opin Infect Dis. 2018;31(5):383–92. 10.1097/QCO.0000000000000485. 30124496 10.1097/QCO.0000000000000485

[4] Brunetti E, Kern P, Vuitton DA. Expert consensus for the diagnosis and treatment of cystic and alveolar echinococcosis in humans. Acta Trop. 2010;114(1):1–16. 10.1016/j.actatropica.2009.11.001. 19931502 10.1016/j.actatropica.2009.11.001

[5] European Food Safety Authority. The European Union One Health 2023 Zoonoses report. EFSA J. 2024;22(12):9106. 10.2903/j.efsa.2024.9106. 10.2903/j.efsa.2024.9106 PMC11629028 39659847

[6] Richter J, Esmann L, Lindner AK, Trebesch I, Equihua-Martinez G, Niebank M, et al. Cystic echinococcosis in unaccompanied minor refugees from Afghanistan and the Middle East to Germany, July 2016 through June 2017. Eur J Epidemiol. 2019;34(6):611–2. 10.1007/s10654-019-00492-8. 30739267 10.1007/s10654-019-00492-8

[7] Yan J, Li Y, Chen Q, Ye X, Li J. Rare orbital cystic lesions in children. J Craniomaxillofac Surg. 2015;43(2):238–43. 10.1016/j.jcms.2014.11.013. 25530304 10.1016/j.jcms.2014.11.013

[8] Ozdemir HB, Ozdek S, Ozkan K, Akyurek N. Primary subretinal hydatid cyst presenting as leukocoria in a child. Retin Cases Brief Rep. 2024;18(2):221–4. 10.1097/ICB.0000000000001347. 36026707 10.1097/ICB.0000000000001347

[9] Benazzou S, Arkha Y, Derraz S, El Ouahabi A, El Khamlichi A. Orbital hydatid cyst: review of 10 cases. J Craniomaxillofac Surg. 2010;38(4):274–8. 10.1016/j.jcms.2009.10.001. 19897382 10.1016/j.jcms.2009.10.001

[10] Papathanassiou M, Petrou P, Zampeli E, Vergados I, Paikos P. Disseminated hydatid disease in a child: albendazole treatment of orbital cyst. Eur J Ophthalmol. 2008;18(6):1034–6. 10.1177/112067210801800634. 18988186 10.1177/112067210801800634

[11] Kahveci R, Sanli AM, Gurer B, Sekerci Z. Orbital hydatid cyst. J Neurosurg Pediatr. 2012;9(1):42–4. 10.3171/2011.10.PEDS11126. 22208319 10.3171/2011.10.PEDS11126

[12] Lentzsch AM, Gobel H, Heindl LM. Primary orbital hydatid cyst. Ophthalmology. 2016;123(7):1410. 10.1016/j.ophtha.2016.02.042. 27342324 10.1016/j.ophtha.2016.02.042

[13] Chtira K, Benantar L, Aitlhaj H, Abdourafiq H, Elallouchi Y, Aniba K. The surgery of intra-orbital hydatid cyst: a case report and literature review. Pan Afr Med J. 2019;33:167. 10.11604/pamj.2019.33.167.18277. 31565128 10.11604/pamj.2019.33.167.18277 PMC6756819

[14] Kumar V, Surve A, Biswal D, Verma N, Kashyap S, Venkatesh P, et al. Intra-ocular hydatid cyst in a child: a rare presentation. Eur J Ophthalmol. 2023;33(3):NP70–4. 10.1177/11206721221079481. 35166612 10.1177/11206721221079481

[15] Alabdullah MN, Awad A, Alabdullah H. Total resection of Intraorbital Hydatid Cyst by using Lynch approach: a case report. Ear Nose Throat J. 2025;104(1_suppl):120S-S123. 10.1177/01455613221113802. 35996338 10.1177/01455613221113802

[16] Awad AA, Mohammad AEA. A simple transconjunctival technique for the management of intraconal orbital hydatid cyst. Indian J Ophthalmol. 2025;73(2):249–52. 10.4103/IJO.IJO_756_24. 39297476 10.4103/IJO.IJO_756_24 PMC11991541

[17] Saravi SS, Sabouni F, Arefidoust A, Yaftian R, Heydarzadeh S, Rajabi MT. An orbital hydatid cyst involving inferior rectus muscle: a case report. Orbit. 2016;35(2):109–12. 10.3109/01676830.2015.1099705. 26905024 10.3109/01676830.2015.1099705

[18] Cruzado-Sanchez D, Salas-Diaz S, Tellez WA, Maquera-Torres G, Serpa-Frias S. [Primary orbital cystic tumor: A case of hydatidosis in a child]. Rev Peru Med Exp Salud Publica. 2017;34(3):560–3. 10.17843/rpmesp.2017.343.2809. 29267783 10.17843/rpmesp.2017.343.2809

[19] Yurt A, Secer M, Selcuki M. Large primary intraorbital hydatid cyst. Childs Nerv Syst. 2011;27(5):693–5. 10.1007/s00381-011-1412-2. 21369785 10.1007/s00381-011-1412-2

[20] Gokhale SK, Sane VD, Ramanojam S, Gadre PK, Gadre KS, Deshmukh SD. Ipsilateral keratoconus associated with long-standing primary hydatid cyst of the orbit. J Craniofac Surg. 2012;23(4):e344–7. 10.1097/SCS.0b013e3182564f13. 22801177 10.1097/SCS.0b013e3182564f13

[21] Handor H, Bencherif MZ. [Orbital hydatid cyst: non exceptional cause if exophthalmia in Morocco]. Pan Afr Med J. 2013;15:147. 10.11604/pamj.2013.15.147.3167. 24396553 10.11604/pamj.2013.15.147.3167 PMC3880820

